# A Portal-Based Intervention (PATTERN) Designed to Support Medication Use Among Older Adults: Feasibility and Acceptability Study

**DOI:** 10.2196/71676

**Published:** 2025-04-24

**Authors:** Allison Pack, Stacy C Bailey, Rachel O'Conor, Evelyn Velazquez, Guisselle Wismer, Fangyu Yeh, Laura M Curtis, Kenya Alcantara, Michael S Wolf

**Affiliations:** 1 Division of General Internal Medicine, Feinberg School of Medicine, Northwestern University, 750 North Lake Shore Drive, 10th Floor, Chicago, IL, 60611, United States, 1 312-503-0274

**Keywords:** older adults, multiple chronic conditions, polypharmacy, primary care, medication adherence, patient portal

## Abstract

**Background:**

Poor medication adherence among older adults with multiple chronic conditions and polypharmacy is a public health concern stemming from distinct challenges. Prior interventions have largely used a one-size-fits-all approach or resource-intensive approaches inappropriate for busy primary care clinics.

**Objective:**

To address this, Phenotyping Adherence Through Technology-Enabled Reports and Navigation (PATTERN) was adapted from prior work. PATTERN is a portal-based intervention for monitoring self-reported medication adherence challenges among older adults in primary care. This study sought to implement and evaluate PATTERN’s feasibility and acceptability.

**Methods:**

We conducted a patient randomized study with a posttest design. Primary care physicians at the participating health center were informed of the study, and approval was obtained to contact their patients. Patient eligibility included being aged 60 years or older, having prescription medications for ≥8 chronic conditions, and an upcoming visit with a physician who had provided approval. Potentially eligible patients were identified using an electronic health record query, and a research coordinator phoned them to confirm eligibility, assess interest, obtain consent, and conduct enrollment. Randomization occurred following enrollment. Those randomized to PATTERN received a medication adherence assessment in their patient portal accounts several days ahead of their visit. The assessment identified whether a patient was experiencing a medication adherence challenge, and if so, the type (cognitive, psychological, medical, regimen-related, social, or economic). Identified challenges were sent to the patient’s primary care physician. Assessment delivery several days ahead of a visit was thought to offer sufficient time for patients to complete it and clinicians to review any challenges. Approximately 2 weeks after visits, the coordinator recontacted participants to conduct posttest interviews. This ensured clinicians had sufficient time to respond to challenges during or after visits. Posttest interviews measured the self-reported use of the portal, demographic and health characteristics, and for those randomized to PATTERN, intervention satisfaction. Self-reported data were captured in REDCap and analyzed descriptively. Electronic health record data were also analyzed descriptively to objectively identify feasibility, that is, whether intervention arm participants completed the PATTERN assessment.

**Results:**

We enrolled 64 participants (32 received usual care, and 32 received intervention). Most were female (66%, 42/64), not Hispanic or Latino (94%, 60/64), and identified as White (58%, 37/64). The average (SD) age was 75 (6.8) years. Most participants (80%) self-reported using the patient portal ≥12 times per year. However, electronic health record data revealed that less than half of all participants randomized to PATTERN (47%, 15/32) completed the medication adherence assessment. Of those who remembered completing it, 60% (3/5) were very satisfied with the experience and 20% (1/5) were a little satisfied.

**Conclusions:**

PATTERN has the potential for use with older primary care patients experiencing multiple chronic conditions and polypharmacy. Yet, further adaptation is needed to ensure recipients access their patient portal accounts and complete assessments.

## Introduction

Older adults with multiple chronic conditions and polypharmacy are at increased risk of suboptimal medication adherence [1,2], resulting in poor treatment outcomes, adverse drug events, and reduced quality of life [3]. In fact, poor medication adherence in this population is considered a substantial public health concern, impacting both morbidity and financial strain [4].

Barriers to medication adherence, or taking medication as prescribed, are well known in older adults with multiple chronic conditions and polypharmacy and comprise distinct challenges that can be mapped to the model of medication self-management; these include challenges classified as cognitive, psychological, medical, regimen-related, social, or economic [5]. Yet, the vast number of interventions designed to address medication adherence challenges do so using a “one-size-fits-all approach,” as opposed to a more tailored approach, targeting the distinct challenges experienced by individual patients [6]. Furthermore, interventions that do tailor approaches are often resource-intensive, requiring clinical pharmacists, community health workers, or other individuals tasked with providing intensive one-on-one sessions [6-8]. These resource-intensive interventions and activities are not always available or appropriate for busy primary care settings. Yet, primary care remains the front line of health care for most older adults in the United States [9].

To address this, we created PATTERN: Phenotyping Adherence Through Technology-Enabled Reports and Navigation. Adapted from a previous study by our team [10,11], PATTERN uses the patient portal to identify and classify medication adherence challenges for review and resolution by a primary care physician or designated care team. It was designed to be low-cost, sustainable, and scalable using existing electronic health record (EHR) technology. We conducted a small feasibility study of this intervention to determine its feasibility and acceptability among older primary care patients with multiple chronic conditions and polypharmacy.

## Methods

### Study Design

We conducted a patient randomized feasibility study, using a posttest design to examine the effects of PATTERN. This paper largely presents data from the intervention arm pertaining to the feasibility and acceptability of PATTERN.

### Eligibility Criteria, Recruitment, and Setting

We first sought approval, via email, from physicians affiliated with any primary care clinic (including geriatrics) within the participating academic health center in Chicago, IL, to contact their patients for recruitment into PATTERN. Patients were eligible to participate if they were aged 65 years or older, spoke English as their primary language, self-reported contending with at least two chronic health conditions, were prescribed eight or more medications they were primarily responsible for taking, and had a visit scheduled within the next 1 week with the physician who had provided contact approval. For intervention purposes, individuals also needed to have access to the internet, an active email address, and a patient portal account. Exclusion criteria included having participated in our prior, formative research or having any severe, uncorrectable visual, hearing or cognitive impairments that would have precluded study consent or participation.

Potentially eligible patient participants were identified using an EHR query, phoned by a trained research coordinator, informed about the study, and screened for eligibility. Those who met all eligibility criteria and expressed an interest in participating then provided electronic informed consent prior to enrollment. The research coordinator scheduled remote posttest interviews with enrolled participants; these occurred approximately 2 weeks after their scheduled clinic visit.

### Randomization

Participants were randomized one-to-one to usual care or the PATTERN intervention using random block sizes of two and four, stratified by clinic type (geriatrics or other primary care clinic). The allocation table was created using SAS PROC PLAN and implemented using REDCap software [12]. Randomization took place immediately following enrollment (Multimedia Appendix 1). The purpose was to be able to compare differences between those randomized to PATTERN and those in usual care.

### Intervention Activities

Participants who were randomized to the PATTERN intervention were sent an email 2 to 3 days ahead of their clinic visit. The email instructed them to log into their patient portal accounts to complete a brief medication adherence assessment prior to their upcoming primary care visit. Those who logged in were instructed to begin the two-tiered assessment adapted from a prior study [10]. The first three questions identified if the participant was experiencing any challenges taking their medications as prescribed. Specifically, these asked: (1) During the past month, did you decide to stop taking any of your medicines without telling your doctor? (2) During the past month, did you decide to take more or less of any of your medicines than you were supposed to without telling your doctor? (3) Are you having any problems with your medicines? Those who endorsed at least one adherence challenge were asked to respond to a subsequent set of 18 questions. These questions, informed by the model of medication self-management, were designed to “phenotype” or identify the distinct type of medication adherence challenge a participant might have been experiencing. Challenges could be classified as cognitive (eg, forgetfulness), psychological (eg, health literacy, depression, or motivation), medical (eg, acute illness), regimen-related (eg, side effects or complex dosing schedules), social (eg, transportation or support), and economic (eg, cost) [5].

Any identified and “phenotyped” challenges were designed to be automatically and securely forwarded, via the EHR, to the participant’s primary care physician with whom they had an upcoming clinic visit. The intent was to ensure physicians received relevant and actionable data from patients regarding any medication challenges they might have been experiencing. This information could subsequently inform the scheduled visit or referral to available support services such as social workers or clinical pharmacists, depending on clinic-specific resources.

### Data Collection Activities

Data were collected between March and August 2024. Remote, structured study interviews were held with individual participants approximately 2 weeks after each participant’s regularly scheduled clinic visit. The research coordinator phoned participants at previously agreed-upon times and administered a brief questionnaire. Data were captured using the REDCap software [12]. On average, it took participants 20 minutes to complete the questionnaire, and all participants were compensated US $50 for their time.

### Measures

#### Participant Characteristics

All participants reported their age, sex, race, and ethnicity. Perceived health status was measured using the single-item patient-reported measurement information system (PROMIS) global health measure that asks participants to rate their health from poor to excellent using a 5-point Likert scale [13]. Depression was captured using the validated 6-item PROMIS depression measure, with higher scores representing greater symptom burden [13]. Patient activation was captured using the validated 10-item Consumer Health Activation Index (CHAI) [14]. Finally, the use of the patient portal was examined by asking participants how often they use their portal accounts and for what purposes. This self-reported information was supplemented by EHR data identifying the completion of any portal questionnaire in the past year.

#### Intervention Feasibility

We used data captured from self-reporting and from the EHR to examine intervention feasibility among participants randomized to PATTERN. Specifically, we were interested in identifying (1) the number of adherence assessments received (opened) among those sent; (2) the number of adherence assessments completed among those received; and (3) the number of participants who spoke with their clinical care teams about the adherence assessments in those who completed them.

#### Intervention Acceptability

Participants who completed the intervention were asked how satisfied they were with the PATTERN questionnaire. Response options ranged on a 4-point Likert scale from “not at all satisfied” to “very satisfied.”

### Analysis

Descriptive statistics were used to examine participant characteristics, overall and by study arm. All feasibility and acceptability data were also assessed using descriptive statistics in the intervention arm only. Analyses were conducted using SAS 9.4 software.

### Ethical Considerations

All study procedures were approved by the Northwestern University’s Institutional Review Board (STU00217555). All participants provided electronic informed consent prior to study participation and were compensated US $50 for their time. Data presented herein have all been deidentified.

## Results

### Participant Characteristics

Participant characteristics are presented in Table 1. In brief, a total of 64 patient participants were enrolled. The average (SD) age was 75 (6.8) years. Most indicated they were female (66%, 42/64), non-Hispanic (94%, 60/64), White (58%, 37/64), and in good or better health (64%, 41/64). A total of 16% (10/64) were classified as having mild or moderate depression, and 38% (24/64) as having low patient activation. A large majority of participants (80%, 51/54) reported they used the patient portal more than 12 times a year, and that when they access the portal, they view laboratory results (98%, 63/64), confirm appointments (89%, 57/64), and send messages to their care teams (91%, 58/64). Actual use, assessed via EHR data, revealed that most patients (72%, 46/64) had previously (within the past year) completed any type of assessment in the patient portal. No significant differences in participant characteristics were observed between study arms.

**Table 1.** Descriptive statistics of participant characteristics and technology use, overall and by study arm, captured via self-reporting and the electronic health records between March and August 2024.

Characteristics	Overall (n=64)	Usual care (n=32)	Intervention (n=32)
Gender, n (%)			
Male	22 (34.4)	12 (37.5)	10 (31.3)
Female	42 (65.6)	20 (62.5)	22 (68.8)
Age, years			
Mean (SD)	75.0 (6.8)	75.1 (7.5)	74.9 (6.1)
Median (range)	74.5 (66-93)	74 (66‐93)	74.5 (66-90)
Ethnicity, n (%)			
Not Hispanic or Latino	60 (93.8)	29 (90.6)	31 (96.9)
Hispanic or Latino	1 (1.6)	1 (3.1)	N/A^a^
Refused	3 (4.7)	2 (6.3)	1 (3.1)
Race, n (%)			
White	37 (57.8)	19 (59.4)	18 (56.3)
Black or African-American	23 (35.9)	11 (34.4)	12 (37.5)
Other	3 (4.7)	2 (6.3)	1 (3.1)
Refused	1 (1.6)	N/A	1 (3.1)
Health status, n (%)			
Excellent	1 (1.6)	1 (3.1)	N/A
Very good	11 (17.2)	3 (9.4)	8 (25.0)
Good	29 (45.3)	17 (53.1)	12 (37.5)
Fair	20 (31.3)	10 (31.3)	10 (31.3)
Poor	3 (4.7)	1 (3.1)	2 (6.3)
PROMIS^b^ depression, n (%)			
Within normal limits	54 (84.4)	29 (90.6)	25 (78.1)
Mild or moderate	10 (15.6)	3 (9.4)	7 (21.9)
CHAI,^c^ n (%)			
Low	24 (38.1)	9 (28.1)	15 (48.4)
Moderate	27 (42.9)	16 (50.0)	11 (35.5)
High	12 (19.1)	7 (21.9)	5 (16.1)
Patient portal use			
Self-reported frequency, n (%)			
Never	2 (3.1)	N/A	2 (6.3)
1 to 6 times per year	1 (1.6)	1 (3.1)	N/A
7 to 12 times per year	10 (15.6)	4 (12.5)	6 (18.8)
More than 12 times per year	51 (79.7)	27 (84.4)	24 (75.0)
Self-reported activities, n (%)			
Requesting appointments	40 (62.5)	22 (68.8)	18 (56.3)
Viewing laboratory results	63 (98.4)	32 (100.0)	31 (96.9)
Refilling prescriptions	51 (79.7)	26 (81.3)	25 (78.1)
Sending a message to the care team	58 (90.6)	29 (90.6)	29 (90.6)
Paying bills	32 (50.0)	13 (40.6)	19 (59.4)
Confirming appointments	57 (89.1)	29 (96.6)	28 (87.5)
Other	2 (3.1)	1 (3.1)	1 (3.1)
Actual use, n (%)			
Any questionnaire completion in the past year	46 (71.9)	25 (78.1)	21 (65.6)

a N/A: not applicable.

b PROMIS: patient-reported measurement information system.

c CHAI: Consumer Health Activation Index.

### Intervention Feasibility and Acceptability

Our assessment of intervention feasibility revealed that 47% (15/32) of participants in the intervention arm completed the adherence assessments that were sent in their patient portal accounts. Of those, only 1 indicated they had any adherence challenges, according to EHR data, though this participant self-reported they did not remember having any challenges. Similarly, when asked by the research coordinator, 75% (24/32) of intervention arm participants self-reported they were unaware they had received an email or portal message asking them to complete the PATTERN medication adherence assessment, and therefore did not complete it. When probed for an explanation, most indicated they had accessed their email but did not notice the request (71%, 17/24). Of those who self-reported receiving the request (25%, 8/32), all but 1 found the adherence assessment to be somewhat or very easy to access, and most (63%, 5/8) reported they completed it. Of the 5 who said they completed the survey, 3 (60%) were very satisfied with the experience, 1 (20%) was a little satisfied, and 1 (20%) did not reply. Moreover, although none of these participants reported having any challenges with their medications, 2 revealed they had discussed the medication assessment and medication taking strategies with their clinical care teams and were satisfied with that conversation. Participants who accessed but did not complete the assessment (37%, 3/8), revealed it was not because they encountered challenges accessing or responding to it; rather, they indicated they did not want to complete it at that time.

## Discussion

This small patient randomized feasibility study examined the feasibility and acceptability of the PATTERN intervention among older adults with multiple chronic conditions and polypharmacy from a large academic medical center in Chicago, IL. Results revealed that the previsit delivery of portal-based assessments has the potential to enhance patient-provider communication about medication adherence in primary care, as most of our sample self-reported being active users of the patient portal and had a record of a completed portal assessment in the EHR in the last year. This is an important finding as our studies have previously shown that while highly resource-intensive interventions can increase medication adherence among older adults, less-intensive options, such as portal-based assessments, are needed for busy primary care clinics [7]. This finding also has far-reaching implications, as health systems are increasingly relying on patient-reported outcomes collected via the patient portal [15-17].

However, less than half of all intervention arm participants completed the PATTERN adherence assessment ahead of their clinical care visit, as instructed. As such, the current approach likely requires additional nudges or supports to ensure all recipients are able to access and complete the assessments in a timely manner. This could include (1) information explaining how to know when assessments are available for completion in the patient portal; (2) more tailored information designed to entice recipients to open the portal message; and (3) detailed information on how to complete assessments on time. Prior portal-based studies conducted among older adults have also shown that this population could benefit from usability training [18,19]. Yet beyond a “how-to” training, information on why portal-based assessments are useful has also been identified as a need [20]. Future studies should explore the nudges, supports, or training older adults find acceptable, feasible, and appropriate. Furthermore, it may be that assessment results need to be sent to the patient ahead of their visit. These could be accompanied by a list of result-specific questions (perhaps even populated by artificial intelligence) that patients could ask during their visit [21]. Not only would this list serve as a reminder that they had completed the assessment, but as other studies using question prompt lists have shown, it can further care engagement [22,23].

The few participants in our study who reported completing the PATTERN assessment noted they were largely satisfied with it and the resulting conversations they had with their care teams. Had we conducted posttest interviews immediately following the clinical care visit, more participants would have likely been able to share their experiences and whether they found the intervention to be acceptable. Increasingly, studies are revealing that older adults typically have positive experiences with patient portals and technology-delivered assessments [24,25]

Results from this study need to be considered in light of the study limitations. First, this was a small feasibility study conducted within a single academic health system. It is possible that a larger, more diverse study would have resulted in different findings. However, the purpose of this study was to obtain preliminary data that could inform a larger, more diverse trial. There was also no age limit for participation in this study. It is possible that PATTERN may be more feasible for the “younger” older adults, for example, those individuals who are between the ages of 65 and 75 years. Finally, we may need to account for “proxy users,” other individuals appointed by the patient to access and respond to needs in their patient portal accounts. Although nearly all participants in our study had not officially authorized “proxy use” in their accounts, it is possible they relied on unofficial use. A recent national survey revealed that nearly half of all older adults between the ages of 50 and 80 years have allowed others to use their portal accounts [17]. This might occur if a patient’s caregiver, for example, used the patient’s login information to confirm appointments, update medication information, or use other portal-based features, including assessments [26]. It is possible that participants in the PATTERN intervention arm did not remember completing the medication adherence assessment because their proxy user had completed it for them.

In conclusion, PATTERN, a remote medication adherence monitoring intervention, shows promise for use with older primary care patients experiencing multiple chronic conditions and polypharmacy. However, further adaptation and training are needed to ensure participants are accessing their patient portal accounts in a timely manner ahead of scheduled clinic visits. Results from this small feasibility study may inform other research and clinical activities intending to capture self-reported data from older adults via the patient portal.

## Supplementary material

10.2196/71676 Multimedia Appendix 1 CONSORT flow diagram.

## ABBREVIATIONS

CHAI: Consumer Health Activation Index
EHR: electronic health record
PATTERN: Phenotyping Adherence Through Technology-Enabled Reports and Navigation
PROMIS: patient-reported measurement information system
