# Supplementary figures and images for: A Portal-Based Intervention (PATTERN) Designed to Support Medication Use Among Older Adults: Feasibility and Acceptability Study
Source: JMIR Form Res. 2025 Apr 24;9:e71676. doi: 10.2196/71676 (PMC12045522; doi:10.2196/71676)

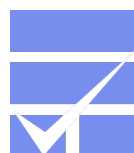

# CONSORT

TRANSPARENT REPORTING of TRIALS

## CONSORT 2010 Flow Diagram

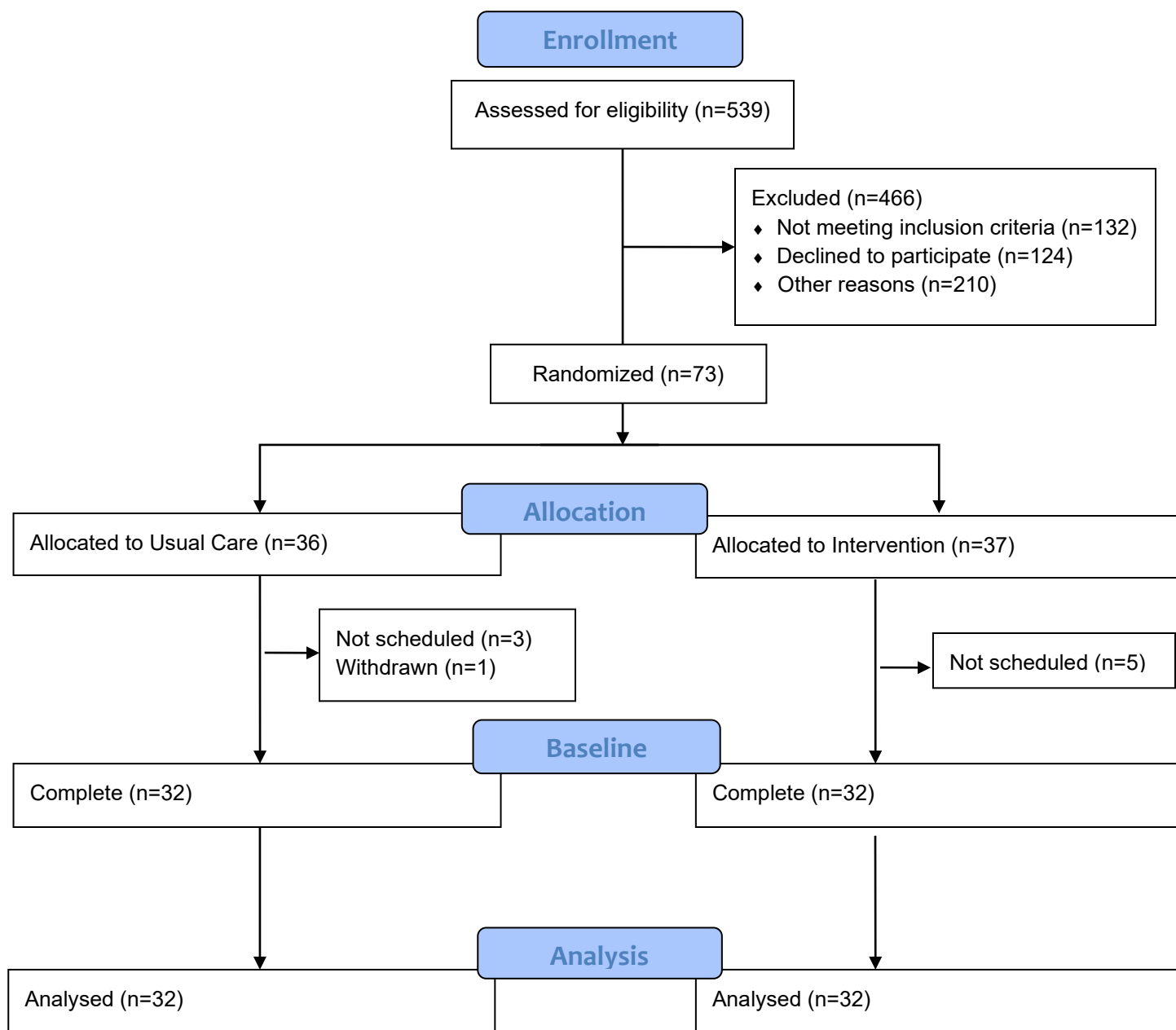

Supplement: Multimedia Appendix 1 [file formative-v9-e71676-s001.pdf]
